# Internet-delivered cognitive behavioural therapy for insomnia disorder in depressed patients treated at an outpatient clinic for mood disorders: protocol of a randomised controlled trial

**DOI:** 10.1186/s12888-022-04492-z

**Published:** 2023-01-27

**Authors:** A. Y. Schotanus, E. Dozeman, S. L. C. Ikelaar, A. van Straten, A. T. F. Beekman, F. van Nassau, J. E. Bosmans, A. van Schaik

**Affiliations:** 1 GGZ inGeest, Specialized Mental Health Care, Amsterdam, The Netherlands; 2 Department of Psychiatry, Amsterdam UMC Location Vrije Universiteit Amsterdam, Boelelaan 1117, Amsterdam, The Netherlands; 3 Amsterdam Public Health, Mental Health Program, Amsterdam, The Netherlands; 4 Department of Health Sciences, Faculty of Science, VU University Amsterdam, Amsterdam Public Health Research Institute, De Boelelaan 1105, 1081 HV Amsterdam, The Netherlands; 5 Department of Clinical-, Neuro- and Developmental Psychology, Faculty of Behavioural and Movement Sciences, VU University Amsterdam, Amsterdam, The Netherlands; 6 Department of Public and Occupational Health, Amsterdam UMC, VU University Amsterdam, Amsterdam Public Health Research Institute, Amsterdam, The Netherlands

**Keywords:** Internet-delivered, Cognitive behavioural therapy for insomnia (CBTi), I-Sleep, Depression, Insomnia disorder (ID), e-health, Randomised controlled trial (RCT), Cost-effectiveness

## Abstract

**Background:**

Major depression is a highly prevalent disorder causing severe personal distress, and high societal costs. Patients with depression often have comorbid insomnia disorder (ID) leading to even worse personal distress and worse treatment outcomes. Recent results from a non-randomised pilot study with internet-delivered Cognitive Behavioural Therapy (CBTi) for Insomnia (I-Sleep) added to regular depression care were promising regarding feasibility and initial effects on insomnia complaints and depression. However, no randomised controlled trial (RCT) has been performed yet to access the (cost-) effectiveness of I-Sleep for depression. Therefore, this protocol article presents the design of an RCT aimed to assess the (cost-) effectiveness of I-Sleep in addition to usual care for depression compared to usual care alone in depressed patients with a comorbid Insomnia Disorder (ID) treated at outpatient clinics for mood disorders.

**Methods /design:**

This is a multi-centre RCT with measurements at baseline and at 3, 6, 9, and 12 months of follow-up. Patients with depression and an ID are randomised to either I-Sleep treatment followed by regular depression care or to regular depression care alone. Our aim is to recruit one hundred and seventy-five patients from multiple outpatient clinics for mood disorders. The primary outcome is the change in depressive symptoms over 12 months of follow-up measured with the Patient Health Questionnaire (PHQ-9). Secondary outcomes are recovery from depression (PHQ-9), insomnia severity (Insomnia Severity Index, ISI), daily functioning (Work and Social Adjustment Scale, WSAS), general quality of life (EuroQol 5-level version, EQ-5D-5L), and societal costs (Adapted versions of the iMTA Productivity Cost Questionnaire, iPCQ and iMTA Medical Cost Questionnaire, iMCQ).

**Discussion:**

We hypothesize that the addition of I-Sleep to usual care will result in a significant improvement in depression treatment outcomes and quality of life as well as a decrease in healthcare and societal costs compared to usual care alone. This study is the first pragmatic RCT evaluating the effectiveness and cost-effectiveness of adding CBTi to usual care for depression.

**Trial registration:**

Netherlands Trial Register (NL8955). Registered on October 6^th^2020. https://trialsearch.who.int/Trial2.aspx?TrialID=NL8955

**Supplementary Information:**

The online version contains supplementary material available at 10.1186/s12888-022-04492-z.

## Background

Major depression is one of the most common mental disorders worldwide, with currently over 264 million people suffering from depression globally [1]. With a lifetime prevalence of 18.7% and a 12-month prevalence of 5.2% in the Netherlands, it is estimated that each year 546,500 people suffer from depression in the Netherlands [2]. Depression is associated with severe functional impairments, and high societal expenditures due to increased healthcare utilization and productivity losses [3–5]. In Europe, the total costs of major depression were estimated to be €92 billion in 2010, of which €38 billion concerning healthcare and patient costs, and €54 billion concern lost productivity costs [6].

Insomnia has a high prevalence as well with estimates for chronic insomnia ranging from 10 to 15% and for transient or occasional insomnia from 25 to 35% in the general adult population [7]. The societal consequences of insomnia are significant and include costs of insomnia treatments, increased health care utilization, decreased workplace productivity, and an increased accident risk [8].

Depression and insomnia often co-occur. It is estimated that of people with insomnia, 40% suffer from depression and that 80% of patients with depression also suffer from insomnia [9]. Moreover, patients with depression and insomnia have lower quality of life [10]. and worse treatment outcomes compared to patients with depression alone [11]. Furthermore, suicidal ideation is more common in this group than among patients with depression alone [11, 12]. Finally, patients who still experience insomnia after successful depression treatment are at higher risk of recurrent depression [13, 14].

Current guidelines for insomnia issued by the Dutch College of General Practitioners (Nederlands Huisartsen Genootschap, NHG) and the European Sleep Research Society recommend that treatment of insomnia should be primarily non-pharmacological, using cognitive behavioural therapy for insomnia (CBT-I) [15]. However, despite disadvantages, such as addiction and undesirable side effects, sleep medication is often prescribed to depressed outpatients with comorbid insomnia. This may be related to the belief held by therapists that symptoms of insomnia will disappear when depression is treated successfully. Another possible reason may be that therapists feel that they are no experts in the treatment of insomnia [16]. As a result, accessibility of CBTi may be limited due to a shortage of experienced therapists [9]. Thus, despite the negative effects of co-occurrence of insomnia and depression, insomnia is hardly ever specifically targeted with CBTi during the treatment for depression in a mental healthcare setting and as a result is often still present (in about 25% of patients) after treatment of depression [17].

Online CBTi has been shown to be effective in a large number of studies [18, 19]. Besides effectively improving insomnia related outcome measures, online CBTi also appears to reduce depressive symptom severity [20, 21]. Moreover, a recent study by Carney, (2017) showed that depression treatment alone can even worsen sleep symptoms [17].

I-Sleep is a guided online CBT intervention developed in the Netherlands that has been tested and found effective in various populations [9, 22, 23]. Recently, I-Sleep was adapted to serve as an add-on to usual care for depressive patients with comorbid insomnia treated in specialized mental healthcare [24]. This non-randomised pilot study by Dozeman, (2019) showed that the addition of I-Sleep to regular depression care resulted in a between-group standardized mean difference in sleep quality of 0.99 (*p* = 0.02) and in depressive symptoms 0.55 (*p* = 0.19) after treatment [24]. Furthermore, this pilot showed the feasibility of implementation of I-Sleep in a large mental healthcare outpatient clinic for mood disorders. Thus, adding I-Sleep to care as usual may improve outcomes in patients who are treated for depression while having comorbid insomnia. However, to date no study has yet examined the effectiveness of adding I-Sleep to regular depression care in a randomised controlled trial in a clinical mental health care setting in the Netherlands. Moreover, despite the profound economic burden associated with depression and insomnia, thus far, no study has assessed cost-effectiveness of adding I-Sleep to depression care as usual.

In this paper, we present the EINSTEIN study protocol (an acronym for: Effectiveness and cost-effectiveness of INternet-based treatment of insomnia in depressed patients Treated at a mental healthcarE outpatient cliINic). The EINSTEIN study is a randomised controlled trial with an economic evaluation alongside to evaluate the effectiveness and cost-effectiveness of adding I-Sleep to usual care for depression. We hypothesize that adding I-Sleep to regular depression treatment will be superior to usual care alone with regards to severity of depression and quality of life. Additionally, we expect that with improved treatment outcomes healthcare and societal costs decrease compared to usual care alone.

## Methods and design

### Study design and aims

This study is a pragmatic multi-centre, randomised controlled trial with an economic evaluation from both a societal and healthcare perspective. The overall objective of the study is to assess the effectiveness and cost-effectiveness of the internet-delivered CBTi treatment program I-Sleep as an addition to usual depression care alone over 12 months. Participants in this study are patients with both depression and insomnia disorder treated at an outpatient clinic for mood disorders in the Netherlands. Furthermore, a process evaluation of implementing I-Sleep in daily clinical practice will be conducted to assess the feasibility of I-Sleep as an integral component of depression treatment. The study protocol, recruitment materials, and measurement methods were approved by the Medical Ethics Committee of the VU University Medical Centre. This trial is conducted according to the principles of the declaration of Helsinki. Likewise, this trial adheres to the SPIRIT guidelines and methodology, see Additional file 1: Appendix A SPIRIT checklist.

### Recruitment

**Fig. 1:**
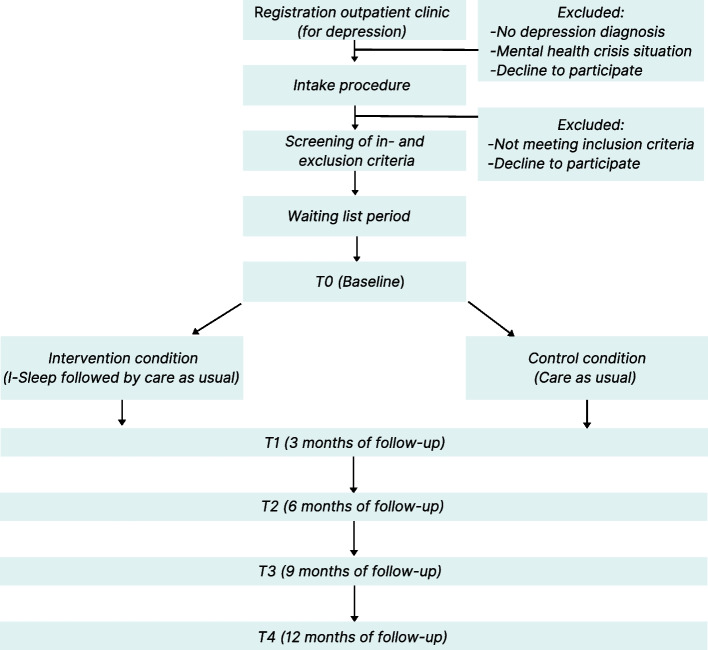
Flowchart of the EINSTEIN study design

Patients are recruited within four mental health outpatient clinics for mood disorders throughout the Netherlands all offering e-Health interventions. Therapists and research assistants on-site are instructed to alert patients with depression and possible comorbid insomnia about the study. Patients who have shown interest in participating in scientific research during intake will be contacted by a research assistant and provided with further information concerning participation in the trial. Patients who consent are screened for eligibility by telephone. The presence of insomnia disorder will be assessed with a self-constructed questionnaire based on the DSM-5 criteria for insomnia disorder. Also, in addition to the clinical judgement of the psychologist at intake, items from the Mini International Neuropsychiatric Interview (MINI) will be administered to confirm the presence of unipolar depression. Eligible patients will be sent an information kit (including information about data storing and handling procedures) and an informed consent form by mail. When the signed informed consent form is returned to the research assistant on site, participants will be randomised into a treatment arm and will receive a link via email to complete the baseline measurement. After providing informed consent, participants can withdraw from the study at any time for any reason without consequences. For a more detailed account of patient flow in this trial see Fig. 1.

### Inclusion and exclusion criteria

In order to be eligible to participate in this study, patients should be between 18 to 75 years old and diagnosed with unipolar major depressive disorder according to the DSM-5 [25] criteria during intake and should be scheduled for treatment for depressive disorder at one of the participating mental healthcare outpatient clinics. In addition, all patients must fulfil the DSM-5 criteria for an insomnia disorder [25] during the telephone screening. Exclusion criteria are, insufficient command of the Dutch language (I-Sleep is only available in Dutch) working night shifts, sleep-related disorders other than insomnia (e.g., sleep apnoea) previously diagnosed by a physician or other (mental) health care specialist, no daily access to an internet-connected computer and the presence of a mental health crisis. Other comorbid mental disorders or somatic diseases are allowed if they do not provide a sufficient explanation for the existence of insomnia complaints. The use of antidepressant medication and sleep medication is allowed in both treatment arms. All in- and exclusion criteria are assessed during a screening procedure performed by telephone by the research assistant on-site using items from the MINI Diagnostic Interview [26] and a DSM-5 insomnia criteria-based screener designed for this study.

### Randomization, blinding and treatment allocation

After inclusion, participants are randomly assigned to either the intervention group I-Sleep (before the start of the usual care) or the control group (usual care alone) on a 1:1 ratio. Variable block sizes are used. Randomization takes place at the patient level and is stratified by mental health outpatient clinics for mood disorders. An independent researcher has created an allocation scheme using a computerized random number generator (Castor). Randomization will be performed by another independent research assistant who reveals the randomization outcome. Due to the nature of the intervention, blinding of patients, therapists, researchers and research assistants was not possible.

### The investigational treatment: I-Sleep, CBT-I for insomnia

I-Sleep is a guided, internet-based treatment based on CBTi [22, 27, 28]. The I-Sleep program consists of five online sessions comprising text, videos, and exercises. Feedback on the exercises is provided by a trained therapist through the secured online platform. Online sessions are divided by subject addressing: 1) psychoeducation on normal sleep, sleep disorders, and sleep hygiene (i.e., behaviours that can promote or impede sleep), 2) sleep restriction (i.e., restricting time in bed to increase sleep pressure) and stimulus control (i.e., reinforcing the association of the bed with sleeping), 3) rumination and relaxation techniques, 4) cognitive restructuring (i.e., changing common misconceptions about sleep) and 5) relapse prevention. Patients need to monitor their sleep by filling out a sleep diary throughout the intervention and complete the exercises in the digital workbook. The therapy program takes less than 30 min a day. The total duration of the insomnia intervention is five weeks.

The intervention is provided in a blended format, comprising both face-to-face or videoconferencing sessions and online sessions. To promote compliance and establish a positive working relationship between the patient and therapist the first face-to-face (f-t-f) session is scheduled prior to the first online session. A second f-t-f session is scheduled after the second online session to provide additional support during sleep restrictions The third and final f-t-f session is scheduled upon completion of all online sessions. Following the I-Sleep intervention, all participants allocated to the intervention group will receive usual care for depression. Preferably both I-Sleep and the usual depression treatment will be delivered by the same therapist so that cognitive techniques introduced during I-Sleep may be worked through more quickly during depression treatment. However, this is not mandated by the study protocol.

I-Sleep is delivered through a secure web-based online treatment platform (Minddistrict, www.minddistrict.com). The platform is owned by a commercial stakeholder from which specialized mental healthcare outpatient clinics for mood disorders buy services. The internet platforms include a PC interface and mobile application providing patients access to I-Sleep, a digital workbook and a secure communication channel for both the therapists and patients.

All therapists involved will receive extensive training prior to their participation in the trial. Training comprises an online course (e-learning) and an interactive webinar providing information on the study design, insomnia in general, and the use of the I-Sleep program. Additionally, a manual describing various scenarios and suggestions for feedback based on possible response patterns of patients is available for trained therapists which can be used as a reference book. Upon completion of the I-Sleep training, bi-monthly Intervision sessions will be organised by experienced CBTi and I-Sleep therapists. Supervision is provided by a senior therapist. In addition, researchers and research assistants are available for questions and to provide technical support at any time for the duration of the trial.

### Usual care

All patients in this study will receive usual care for depression. Usual care is given according to the multidisciplinary Dutch guideline for depression care and contains (group) psychotherapy and/or antidepressant medication treatment. Therapy and medication use during the trial is monitored. Furthermore, therapists are requested to refrain from CBTi (particularly I-Sleep), but usual care is not restricted in any other way.

### Assessments

Measurements will be taken at baseline (T0) and at 3- (T1), 6- (T2), 9- (T3) and 12-month (T4) follow-up. All measures are completed online; participants will receive a link to the online questionnaires via email. Additionally, a randomly chosen selection of participants and therapists from all participating outpatient clinics for mood disorders will be invited for an in-depth interview by telephone as part of the RE-AIM process evaluation. Exit interviews will be held with participants who discontinue or deviate from intervention protocols. For a detailed overview of the measurements see Table 1.

**Table 1:** Repeated measurements

Questionnaire	T0	T1	T2	T3	T4
Depression severity (PHQ-9)	x	x	x	x	x
Sleep quality (ISI)	x	x	x	x	x
Daily functioning (WSAS)	x	x	x	x	x
Quality of life (EQ-5D-5L)	x	x	x	x	x
Healthcare, patient and family costs (iMCQ)	x	x	x	x	x
Lost productivity costs (iPCQ)	x	x	x	x	x
Process evaluation questions	x	x			x

T0: baseline, T1: 3 months after T0, T2: 6 months after T0, T3: 9 months after T0, T4: 1 year after T0

Online measurements and instruments at different points of assessment. *PHQ-9* Patient Health Questionnaire-9, *ISI* Insomnia Severity Index, *WSAS* Work and Social Adjustment Scale, *EQ-5D-5L* EuroQol, *iMCQ* iMTA Medical Cost Questionnaire, *iPCQ* iMTA Productivity Cost Questionnaire, Process evaluation questionnaire according to RE-AIM framework adjusted to the EINSTEIN study

### Primary outcome: depression severity

The primary outcome is the change in depressive symptoms during one year of follow-up. Depressive symptoms will be assessed with the web-based Patient Health Questionnaire-9 (PHQ-9) at baseline and at 3, 6, 9 and 12 months of follow-up. The PHQ-9 scores each of the nine DSM criteria on a scale from "0" (not at all) to "3" (nearly every day), resulting in a total score ranging between 0 and 27 which is divided into increasing severity categories resulting in the PHQ Depression Severity Index [29]. Recent studies show that the PHQ-9 delivered using web-based technology is a reliable instrument to assess the severity and frequency of depressive symptoms in the previous two-week period [30, 31]

### Secondary outcomes

All secondary outcomes will be assessed online at the same measurement moments, at baseline, and at 3, 6, 9, and 12 months of follow-up using web-based questionnaires. Insomnia severity as measured with the Insomnia Severity Index (ISI) is a secondary endpoint. The ISI is a 7-item brief instrument that measures sleeping difficulties in the past two weeks, which has been validated for online use [32]. Each item is scored on a Likert scale from 0 to 4, comprising topics regarding sleeping patterns, interference with daily life and distress caused by an impaired sleeping pattern [32].

Daily functioning is assessed using the Work and Social Adjustment Scale (WSAS) which is a scale designed to measure functional impairment due to another cause or affliction [33].

General quality of life is measured using the five-level version of the EuroQol questionnaire (EQ-5D-5L) [34]. The EQ-5D-5L contains five dimensions (mobility, self-care, usual activities, pain/discomfort and anxiety/depression) that are rated using five levels (no problems, slight problems, moderate problems, severe problems and extreme problems) [34]. EQ-5D-5L health states will be converted to utility scores using the Dutch EQ-5D-5L tariff [35], which will then be used to calculate Quality-Adjusted Life-Years.

Costs will be measured from a societal perspective using web-based questionnaires based on the iMCQ and iPCQ. Cost categories that will be included are: 1) healthcare costs (primary and secondary care, complementary care and home care); 2) lost productivity costs (absenteeism from paid and unpaid work, and presenteeism); and 3) patient costs (informal care and other care services paid for by patients themselves). Valuation will be done according to Dutch costing guidelines [36]. For the valuation of health care utilization, lost productivity and informal care Dutch standard costs will be used. Medication use will be valued using prices of the Royal Dutch Society for Pharmacy. Patient and family costs other than informal care will be valued using self-reported prices. For the valuation of absenteeism from paid work, the friction cost approach will be used.

### Process evaluation

Parallel to the RCT, we will conduct a process evaluation using the RE-AIM model to investigate the Reach of different patient groups, Effectiveness on patients’ insomnia, Adoption of I-Sleep amongst therapists, Implementation of I-Sleep amongst therapists as well as fidelity of patients to the I-Sleep e-Health modules, and Maintenance of implementation by therapists [37]. For the development of the implementation plan, we will conduct several interviews with patients, therapists and managers from each mental healthcare outpatient clinic for mood disorders during and at the end of the study period. The interviews will also provide us with insight into facilitating and hindering factors as defined in the model of [38] for implementation and sustainable use of I-Sleep in practice, which can provide suggestions for improvements in the evidence-based multi-faceted strategy. The interviews will be recorded, anonymized and transcribed verbatim. The transcripts will be analysed according to standard methodology using thematic content analysis. In addition, a small number of process evaluation questions is added to the assessments at baseline, and at 3 (after the intervention period) and 12 months of follow-up. Table 2 provides an overview of the research objectives and data collection methods for the process evaluation using the RE-AIM framework.

**Table 2:** Overview of research objectives and data collection methods RE-AIM

Quantitative methods					Qualitative methods			
Research objectives:	Baseline online questionnaire	T1 (3 month follow-up) online questionnaire	T4 (12-month folluw-up) online questionnaire	MindDistrictEPR (electronic patient record)	Exit Interview by telephone with patients opting out	Focus group and/or interviews patients (at T1)	Focus group and/or interviews therapists (at T1)	Interviews managers out patient clinics (at T1)
*Reach*								
1. Evaluating the procedures of recruiting Baseline	X					X	X	X
2. Determining reasons for joining, continuing with or opting out of the I-Sleep program among patients	X				X			
3. Determining patient characteristics e.g. demographics and mental health	X			X				
*Effectiveness*								
1. Evaluating the effect on the I-Slee program on insomnia and depression severity		X						
2. Evaluating the effect of the I-Sleep program among patients		X						
3. Determining satisfaction with the I-Sleep program and provided tools/guidance among therapists		X				X		
4. Determining satisfaction with the I-Sleep program therapists							X	
5. Determining satisfaction with the I-Sleep program and provided tools/guidance among outpatient clinic managers								X
*Adoption*								
1. Determining therapist characteristics e.g. demographics, skills and experience	X			X				
2. Evaluating the procedures of recruiting therapists	X			X			X	X
3. Determining reasons among therapists for joining, continuing with or opting out of using the I-Sleep program	X						X	
4. Evaluating the overall (starting) process of recruiting/adopting I-Sleep by the organisation								X
*Implementation*								
1. Determining treatment fidelity among patients, e.g. completed sessions and assignments		X				X		
2. Determining the degree in which the treatment protocol has been followed by therapists (fidelity and dosage)							X	
3. Determining the use (dosage and fidelity) of provided tools and guidance regarding the I-Sleep program among therapists							X	
*Maintenance*								
1. Evaluating the long-term effects and application of techniques from the I-Sleep program among patients			X					
2. Determining helpful elements in maintaining to apply techniques from the I-Sleep program among patients			X			X		
3. Evaluating the long-term use of the I-Sleep program among therapists							X	
4. Determining helpful elements in maintaining to use the I-Sleep program among therapists and in the outpatient clinic in general							X	X
5. Determining future activities and intention to use the program						X	X	X
*Overall*								
Determining the perceived barriers and facilitators to the adoption, implementation and continuations of the program						X	X	X

Measurements at different points of assessment according to the RE-AIM process evaluation principle for patients, therapists and outpatient managers. Where RE-AIM stands for: Reach, Effectiveness, Adoption, Implementation and Maintenance

### Sample size

The sample size calculation is based on a previously conducted pilot study among depressive patients suffering from insomnia [24]. In this pilot study, the mean PHQ-9 score was 11.5 in the intervention condition (I-Sleep + usual care) and 15.0 in the control condition (usual care alone) at three months of follow-up. Assuming an SD of 7.1, an alpha of 0.05, and a power of 0.80, this results in a total required sample of 130. With four participating mental healthcare outpatient clinics for mood disorders and an Intraclass Correlation Coefficient (ICC) of 0.05, the total required sample size for the current trial is 140. Considering a dropout rate of 20% based on the pilot study, the required sample size is 175. This means all four mental healthcare outpatient clinics for mood disorders ought to include approximately 44 patients in total, i.e., 22 patients per year.

### Statistical analysis

Statistical analyses will be performed according to the intention-to-treat principle and will include a head-to-head comparison of the intervention group with the usual care group. A priori, we decided on a subgroup analysis stratified for the use of sleep medication at baseline. Analyses will be (partly) conducted by two independent researchers to check the validity and correctness of the analyses and it will be documented which software is used over the course of this project.

### Primary outcome measures

To assess the difference in the improvement in depression symptom severity as assessed by the PHQ-9 over time between the I-Sleep group and usual care group, linear mixed models that account for the repeated measures within patients will be used. Three hierarchical levels will be included in the linear mixed models: mental healthcare outpatient clinic for mood disorders, patient and time. The primary effect is described by the coefficient of the time-treatment interaction term. If necessary, the analysis will be adjusted for confounders and/or stratified for effect modifiers.

### Secondary outcome measures

To assess the difference in recovery from depression between the I-Sleep and usual care groups over a period of 12 months after baseline, logistic mixed models will be used to account for repeated measurements within patients. To assess the difference in the improvements in insomnia symptom severity, daily functioning, and quality of life over time between the I-Sleep and usual care groups, linear mixed models will be used to account for repeated measurements within patients. Three hierarchical levels will be included in the logistic and linear mixed models: mental healthcare outpatient clinic for mood disorders, patient and time. The primary effect is described by the coefficient of the time-treatment interaction term. If necessary, the analysis will be adjusted for confounders and/or stratified for effect modifiers.

### Economic evaluation

In the economic evaluation, we will relate the incremental costs of I-Sleep in addition to usual care for depression in comparison with usual care alone to the incremental health effects. We will perform both a cost-effectiveness analysis (CEA) and a cost-utility analysis (CUA) from a societal and healthcare perspective according to Dutch guidelines [39]. The time horizon of the economic evaluation is 12 months, making discounting unnecessary. The following effect measures will be included in the economic evaluation: 1) Severity of depressive symptoms (PHQ-9); 2) Recovery from depression (PHQ-9); 3) Sleep quality (ISI); 4) Quality-Adjusted Life-Years.

Multiple imputation according to the MICE algorithm will be done to impute missing cost and effect data [40]. To estimate cost and effect differences between intervention and control while adjusting for confounders if necessary linear regression analyses will be used in every imputed dataset. Next, Rubin’s rules will be used to pool the results from the different datasets. We will calculate Incremental cost-effectiveness ratios (ICERs) by dividing the difference in the mean total costs between the treatment groups by the difference in mean effects between the treatment groups. To estimate statistical uncertainty around the cost differences and the ICERs bias-corrected and accelerated bootstrapping with 5000 replications will be used. To graphically present uncertainty surrounding the ICERs cost-effectiveness planes will be used. The probability that the intervention is cost-effective in comparison with control for a range of different ceiling ratios, i.e., the decision uncertainty, will be shown using cost-effectiveness acceptability curves [41].

### Data management and quality assurance

The data of each participant will be stored in a case record form (CRF patient data file) on each study site. The CRF is available only to the research team on site and will not be disclosed to a third party. Participants in the EINSTEIN study will be pseudonymised, i.e., each participant will be identified by a four-digit number that contains no identifiable information. The master key connecting names to the code will be safeguarded by the principal investigators on each study site only. All data is collected digitally and de-identified using a four-digit code number via Castor, a certified online electronic data capture system for medical research. Except for the qualitative interviews with patients, therapists, and managers from each mental healthcare outpatient clinic for mood disorders during and at the end of the study period held for process evaluation purposes. Audio records of the interviews will be securely stored at the digital storage facilities of each participating location only for as long as it takes to transcribe the interview anonymously since we intend to publish the pseudonymised transcripts (instead of the audiotapes). Additionally, an administrative database will be used on each study site to ensure timely assessments and log any other relevant research activities. At the end of the trial, once collected, the anonymised interview transcripts, the de-identified baseline data, log files of the internet program, the de-identified database data, log files of research activities, and the follow-up data collected from all mental healthcare outpatient clinics will be stored safely at the Department of Research and Innovation of GGZ inGeest. All data will be processed, backed up periodically, and managed by the data management team of GGZ inGeest. Data will be handled confidentially complying with the General Data Protection Regulation (GDPR) and in accordance with the Dutch Medical Research Involving Human Subjects Act (WMO). Likewise, both Castor and the Minddistrict platform on which the I-Sleep intervention runs, comply with all the applicable laws and regulations, including ICH E6 Good Clinical Practice (GCP), 21 CFR Part 11, EU Annex 11, General Data Protection Regulation (GDPR), HIPAA (US), ISO 9001 and ISO 27001. Following a risk assessment of the EINSTEIN study, installing a data and safety monitoring board was deemed unnecessary considering it is not expected that the I-Sleep intervention in this study will cause additional harm or risk to participants. Documentation on the research project will be archived. Research data of the study will be stored and kept for fifteen years upon completion of the EINSTEIN trial and after the publication of the results, after which they will be destroyed.

### Adverse event reporting

All adverse events (AEs) reported spontaneously by the subject or observed by the investigator or staff will be recorded. Serious adverse events (SAEs) for this study are unforeseen. However, SAEs that might be related to the investigational CBTi treatment defined as suicide or acute aggravation of psychiatric symptoms which necessitates additional intervention, such as manic or psychotic episodes will be reported to the sponsor without undue delay. The sponsor will report the SAEs to the accredited METC according to its requirements. SAEs will be followed until they have abated, or until a stable situation has been reached. Given the nature of the investigational treatment, no other serious adverse events directly related to the I-Sleep program are expected.

### Data dissemination

Data will first be analysed by the researchers involved in the project. After a stipulated embargo period the depersonalised data, statistical code, and study protocol will be made accessible for further research and verification upon reasonable request (including a data-analysis plan) and after permission from a (to be installed) steering committee. GGZ inGeest will process the collected data and will disclose both positive and negative findings unreservedly. Results from the EINSTEIN study will be submitted for publication to peer-revied scientific journals and disseminated through presentations at scientific conferences. Findings will be communicated with participants through the websites of participating outpatient clinics for mood disorders and the Dutch Depression Association.

## Discussion

This study is to our knowledge, the first to investigate the effectiveness and cost-effectiveness of adding I-Sleep to care as usual in a population receiving treatment for unipolar depression at several outpatient clinics for mood disorders within the Netherlands. Combining both quantitative and qualitative measures will result in a thorough overview of the effectiveness, cost-effectiveness, and feasibility of I-Sleep in depression treatment. This will allow us to not only provide evidence on the effectiveness, and cost-effectiveness of I-Sleep, but through the RE-AIM process evaluation to also reflect on helping and hindering factors during the implementation process in clinical practice.

Results from this study will be important for several stakeholders. First, if the addition of I-Sleep to usual care of patients with both depression and insomnia proves to be effective, this will improve the treatment of depression in general. Therapists may benefit from the easy-to-use online protocol for the treatment of patients with depression and insomnia, and patients may experience better treatment outcomes. Second, the involvement of mental healthcare providers and representatives of depressed patients ensures that this information will then also be incorporated in clinical treatment decisions for patients with both depression and insomnia. Third, if the cost-effectiveness of I-Sleep has been confirmed, this will be an incentive for mental healthcare institutions to make I-Sleep available for therapists in their organisations on a regular basis. Moreover, this is also expected to lead to reduced societal costs related to depression.

Additionally, an advantage of the I-Sleep intervention over regular CBTi is that it has a relatively low-threshold for patients to start with insomnia treatment, since they can work on treating their insomnia from the comfort of their own homes. Additionally, for therapists, the time investment needed to be trained in I-Sleep is small. I-Sleep builds on what is already known by experienced CBT therapists and less experienced therapists (e.g., general practitioners, nurse practitioners and experts by experience) can be trained in I-Sleep relatively quickly. With this small time-investment in training, I-Sleep also responds to the current work pressure and the general shortage of trained psychologists within the mental healthcare system.

Equally important, this RCT is based on a non-randomised pilot study within one outpatient mental healthcare clinic for mood disorders in which I-Sleep for depression proved to be effective. However, the findings also showed room for improvement in the applicability of I-Sleep in clinical practice. The ‘I-Sleep for depression protocol’ for this RCT was adapted based on the results of this pilot study to facilitate implementation in clinical practice and to reduce the drop-out rate of participants. The following adjustments were made. First, during the pilot study I-Sleep was offered simultaneously with usual care, but this was too much of a burden for the participants. I-Sleep is now offered as part of the depression treatment; the treatment starts with I-Sleep and, after completing the I-Sleep program, treatment continues according to the care-as-usual principle if necessary. We believe that this adjustment will procure a higher percentage of participants finishing the entire I-Sleep program. Secondly, during this RCT I-Sleep is preferably given by the same clinician as the subsequent usual depression treatment. Lastly, we have chosen to offer I-Sleep as a guided intervention instead of entirely as a self-help module. This may increase the acceptance and effectiveness of the treatment offered in people with depression [42].

Besides, many of the available studies on the effect of I-Sleep on insomnia have been conducted in the general population or in general practice patients with sleep problems. We are conducting research with I-Sleep in clinical practice among a population that is not yet well studied but experiences a huge suffering due to (clinically diagnosed) insomnia and depression. Which means that we will soon be able to present results that are relevant and generalizable to clinical practice, where the patients with these problems are found.

Needless to say, as in all studies, the scope of our design is limited. Within the current study design, we recruit patients from institutions that voluntarily applied for participation or with which good contacts already existed. This may cause a selection and/or convenience bias. It may be that these institutions are (more than others) already focused on treating patients with depression for insomnia-related symptoms as well. The contrast between the experimental condition and care as usual could therefore be smaller, which could lead to overly conservative estimates of the effect of I-Sleep.

Likewise, due to a study protocol with randomisation and control group, we may experience a limitation in the number of patients that would want to participate. Patients or “potential participants” find it difficult to understand that only 50% of the participants will be treated with I-Sleep and that this will be determined by randomization. At the same time, some patients insist on starting treatment with the regular depression care immediately and this is not possible when randomised to the I-Sleep condition. The randomisation procedure therefore possibly complicates the recruitment of potential participants.

In summary, adding guided online CBTi to usual care for depression in outpatient clinics for mood disorders might be an effective and cost-effective alternative to usual care alone. This study will evaluate the (cost) effectiveness of I-Sleep for depression while simultaneously assessing the feasibility of implementation of I-Sleep in primary and secondary mental healthcare.

## Trial status

The EINSTEIN study protocol has been approved by the Medical Ethics Committee on September 4^th^ in 2020 (protocol version 6 is currently active). At the time of submission of this manuscript the inclusion phase of the trial is ongoing. The first participants are included, others are being recruited at multiple outpatients mental health clinics in the Netherlands. It is expected that participants will be recruited until January 2024 and data collection will be completed in January 2025.

## Supplementary Information

**Additional file 1.** 

## Data Availability

Results from the EINSTEIN study based on individual participant-level data (IPD) will be shared after de-identification and submitted for publication to peer-revied scientific journals and disseminated through presentations at scientific conferences. Data will first be analysed by the researchers involved in the project. After a stipulated embargo period the depersonalised data, statistical code, and study protocol will be made accessible for further research and verification upon reasonable request (including a data-analysis plan) and after permission from a steering committee of the Amsterdam UMC, location VUmc.

## Abbreviations

AEs: Adverse Events
CBT: Cognitive Behavioural Therapy (CBT)
CBTi: Cognitive Behavioural Therapy for Insomnia
CEA: Cost-effectiveness Analysis
CRF: Case Report Form
CUA: Cost-Utility Analysis
DSM-5: Diagnostic and Statistical Manual of Mental Disorder-5
DSMB: Data safety and monitoring board
EINSTEIN: Acronym for Effectiveness and cost-effectiveness of INternet-based treatment of insomnia in depressed patients Treated at a mental healthcarE outpatient clinic
EQ-5D-5L: EuroQol 5-level version
GDPR: General Data Protection Regulation
HIPAA: Health Insurance Portability and Accountability Act
IC: Informed Consent
ICC: Intraclass Correlation Coefficient
ICER: Incremental Cost-effectiveness Ratio
ICH E6 GCP: International Council for Harmonisation E6 Good Clinical Practice
ID: Insomnia Disorder
IP: Individual Patient Data
iMCQ: IMTA Medical Cost Questionnaire
iPCQ: IMTA Productivity Cost Questionnaire
ISI: Insomnia Severity Index
METC: Medical research ethics committee (MREC); in Dutch: medisch-ethische toetsingscommissie (METC)
MICE: Multiple Imputation by Chained Equations
MINI: Mini International Neuropsychiatric Interview
PHQ-9: Patient Health Questionnaire-9
QALY: Quality Adjusted Life Years
RCT: Randomised Controlled Trial
RE-AIM: Acronym for Reach, Effectiveness, Adoption, Implementation and Maintenance
SAEs: Serious Adverse Events
UMC: University Medical Centers
VUmc: VU University Medical Center
WMO: Medical Research Involving Human Subjects Act
WSAS: Work and Social Adjustment Scale
